# Public engagement on global health challenges

**DOI:** 10.1186/1471-2458-8-168

**Published:** 2008-05-20

**Authors:** Emma RM Cohen, Hassan Masum, Kathryn Berndtson, Vicki Saunders, Tom Hadfield, Dilzayn Panjwani, Deepa L Persad, Gunjeet S Minhas, Abdallah S Daar, Jerome A Singh, Peter A Singer

**Affiliations:** 1 McLaughlin-Rotman Centre for Global Health, University Health Network and University of Toronto, Canada

## Abstract

**Background:**

Experience with public engagement activities regarding the risks and benefits of science and technology (S&T) is growing, especially in the industrialized world. However, public engagement in the developing world regarding S&T risks and benefits to explore health issues has not been widely explored.

**Methods:**

This paper gives an overview about public engagement and related concepts, with a particular focus on challenges and benefits in the developing world. We then describe an Internet-based platform, which seeks to both inform and engage youth and the broader public on global water issues and their health impacts. Finally, we outline a possible course for future action to scale up this and similar online public engagement platforms.

**Results:**

The benefits of public engagement include creating an informed citizenry, generating new ideas from the public, increasing the chances of research being adopted, increasing public trust, and answering ethical research questions. Public engagement also fosters global communication, enables shared experiences and methodology, standardizes strategy, and generates global viewpoints. This is especially pertinent to the developing world, as it encourages previously marginalized populations to participate on a global stage. One of the core issues at stake in public engagement is global governance of science and technology. Also, beyond benefiting society at large, public engagement in science offers benefits to the scientific enterprise itself.

**Conclusion:**

Successful public engagement with developing world stakeholders will be a critical part of implementing new services and technologies. Interactive engagement platforms, such as the Internet, have the potential to unite people globally around relevant health issues.

## Background

The importance of engaging the public on risks and benefits of science and technology (S&T) is widely accepted. Experience with public engagement activities is growing, especially in the industrialized world. However, public engagement in the developing world regarding S&T risks and benefits to explore health issues has received relatively little attention.

The purpose of this paper is to be descriptive. We will briefly review what is known about public engagement, with emphasis on biotechnology-related examples and the developing world. We will then describe our preliminary work on an Internet-based public engagement demonstration project on global water problems and their potential health impact.

## Discussion

### Public Engagement and Related Concepts

We define public engagement as a process that provides people with trustworthy information on key policy issues, elicits their input, and integrates it into decision-making and social action.

The OECD report *Problems and Promises of e-democracy *suggests three levels of public engagement in the government context (a similar distinction holds for public engagement by non-governmental entities) [1]:

1. Information: "a one-way relation in which government produces and delivers information for use by citizens."

2. Consultation: "a two-way relation in which citizens provide feedback to government. It is based on the prior definition by government of the issue on which citizens' views are being sought and requires the provision of information."

3. Active Participation: "a relation based on partnership with government, in which citizens actively engage in the policy-making process. It acknowledges a role for citizens in proposing policy options and shaping the policy dialogue."

We believe active participation where the public is truly empowered and where their input directly influences decisions is appropriate with respect to the risks and benefits of S&T [2].

Public engagement is distinct from community engagement, which focuses on specific communities involved in particular research or activities. Another linked concept, deliberative democracy, refers to both a principle and a set of practices–all based on the idea that legitimate, well-informed decisions grow out of public citizen discussions which balance competing values and policy options [3]. Deliberative polling and citizen deliberative councils use facilitated dialogue of a cross-section of citizens to generate findings and recommendations [4,5].

### Benefits of Public Engagement

Changes to political organisation amid globalisation have empowered individual citizens to influence policy. This idea that citizens have the capacity to contribute to policy and self-governance serves as the rationale for public engagement. The benefits of public engagement include creating an informed citizenry, generating new ideas from the public, increasing the chances of research being adopted, increasing public trust, and answering ethical research questions. There can be significant gains in effectiveness and insight of decisions when the distributed intelligence of the public is combined with that of policymakers [6]. Public engagement also fosters global communication, enables shared experiences and methodology, standardizes strategy, and generates global viewpoints [7]. This is especially pertinent to the developing world, as it encourages previously marginalized populations to participate on a global stage. From public engagement in the developing world, citizens of industrialized countries gain exposure to the challenges faced by the majority of humanity.

One of the core issues at stake in public engagement is global governance of science and technology. Governance is the process of managing affairs either in an organization or society [8]. The function of global governance is to ensure human security and human rights, international rule of law/global ethics, fairness in global distribution, common identity as global citizens and a global voice and channels of participation [9]. One way to realize these functions is through legitimate and democratic representation. The challenge in today's emerging global polity is ensuring representation when the public is not confined to a certain geographical location. To ensure the representation of key stakeholders, namely the poor and marginalized, it has become increasingly important to integrate the issue of public engagement into current debates on global health governance.

Beyond benefiting society at large, public engagement in science offers benefits to the scientific enterprise itself. Given that the world economy is based on capital interests, public engagement can complement market signals in setting the research agenda, resulting in a better match between research priorities and social needs. It can address distrust in science, which has the potential to translate into a lack of support for research [10]. Finally, informed public questioning can probe excessive optimism and help prevent unintended consequences of such optimism [10].

### Principles and Challenges in Public Engagement

Lukensmeyer and Torres discuss principles and challenges for putting public engagement into practice, for both in-person and online venues [11]. They have adopted a set of seven principles: educate participants, frame issues neutrally, achieve diversity, get buy-in from policy makers, support quality deliberation, demonstrate public consensus, and sustain involvement. They also distinguish between "information exchange models" such as public hearings or media broadcast, and "information processing models" such as deliberative forums; one challenge is to scale up the latter methods to have the reach of the former.

Leshner gives a set of lessons for science and public engagement, including, simply: "*Listen*. The most important – and most difficult – lesson to learn is that public engagement involves genuine dialogue, which means both parties must listen and be willing to modify their own positions... We have to mean it when we do it [12]."

The Public Involvement Network for the Canadian Policy Research Network (CPRN) has identified five main challenges in engaging the public [13]. First, it is difficult to measure benefits of engaging citizens. Second, citizens themselves question whether their voices will be listened to and have impact. Third, cost and time can limit the scope of engagement. Fourth, the process of engagement can require time-consuming and continuous monitoring and evaluation. Fifth, appropriate response to public needs may demand institutional reform. As well, broad participation and deep deliberation may be at odds, particularly when differences are strong and feelings run high [14].

In our work with public engagement online platforms, we've found a particular challenge in motivating public interest and involvement. We've learned that building an online platform is not enough; identifying opportunities for users to start projects, keeping barriers to participation low enough for anyone to take part, and launching an aggressive marketing campaign are keys to success for public engagement programs.

### Public Engagement: Two Examples from the Developing World

The literature highlights many examples of public engagement in the developed world but is sparse on recording engagement activities that occur in the developing world. Without meaning to underestimate or discredit the public engagement activities that take place in the developing world, factors that make public engagement effective there are simply less well known. The following two examples illustrate a range of creative public engagement activities in the developing world.

#### Science Communication in Latin America

A number of innovative public engagement methods have been used in Latin America [15]. These have "...included public events in bars and other venues outside the academic circuit, dramas, soap operas, comic books, poetry, games, story-telling, science fairs, and even science-based participation in Peru's parades and Brazil's annual Carnival." The Carnival initiatives aim to "put science on the street" [16]. Interactive science museums, mass media, and online routes have also been used for science communication. Challenges include reaching poorer groups in society, incorporating the full spectrum of relevant issues and uncertainties, and developing initiatives where citizens can openly debate the impact of science on society.

#### Public Understanding of Biotechnology

South Africa is regarded as a biotechnological leader in Africa. One key program, founded in 2003 by SAASTA (South African Association for Science and Technology Advancement) and called the Public Understanding of Biotechnology (PUB), aims to promote an understanding of biotechnology, and to engage the entire South African population in dialogue on its present and future applications [17]. PUB offers a basic biotechnology course, profiles role models from the biotechnology field, conducts surveys of public opinion, provides materials to educators, and provides space on their website for debating key issues. It also offers free posters explaining biotechnology in several of South Africa's eleven national languages, and develops games, crosswords, videos, and puzzles, which promote understanding of biotechnology in an unconventional way. When audience evaluations revealed that the original posters were not stimulating or thought-provoking, PUB modified the approach. PUB has made a concerted attempt to engage the public–an attempt that has arguably been met with some success, achieved amidst a number of challenges facing the diverse and multicultural South African population, including isolated rural areas, the spread of HIV/AIDS, many official languages, widespread illiteracy and poverty, lack of infrastructure and no history of public engagement [18].

We hope that awareness about the importance of public engagement to solve health challenges will increase, especially in the developing world where widespread illness results in health disparities. There is reason to stay hopeful as funding from the Wellcome Trust International Engagement Awards to support public engagement initiatives around health research was recently announced. Other charities and funding organizations are encouraged to follow suit.

### WaterEngage: a public engagement platform on global water challenges

**Figure 1:**
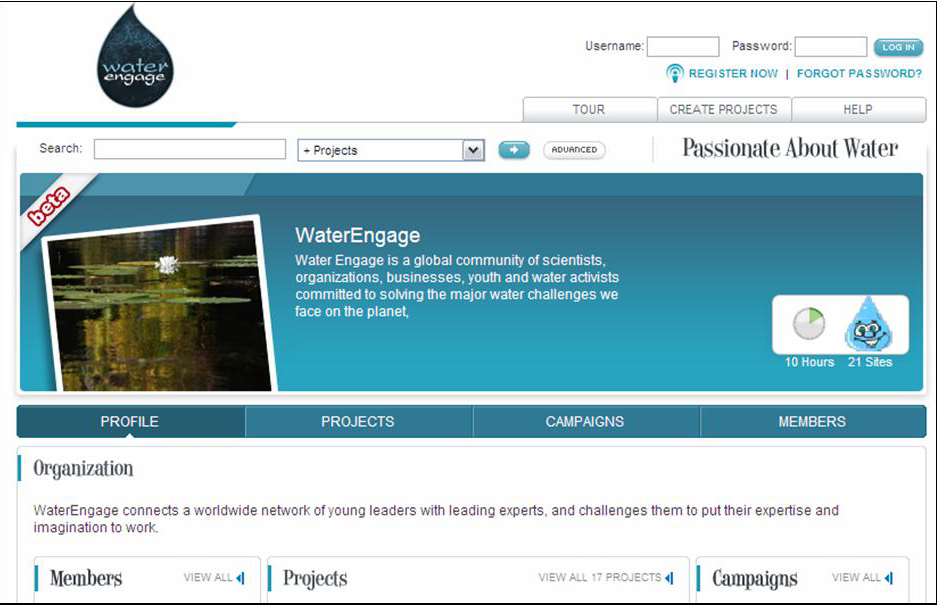
– *WaterEngage *3.0 Homepage. *WaterEngage *is an Internet-based global demonstration project on public engagement.

*WaterEngage *(Figure 1) is an Internet-based global demonstration project on public engagement. The goals of *WaterEngage *are threefold: 1) to raise awareness of the risks and benefits of S&T by educating and empowering youth on one of the greatest public health challenges of our time: global water issues, including water remediation, water scarcity, and water-related diseases; 2) to foster solidarity between youth in developing and industrialized countries through joint projects; and 3) to promote development and funding for Southern-led technology projects that address water challenges. *WaterEngage *aims to enable active participation by the public. The outcome of *WaterEngage *is attainment of a critical mass of users and building of a virtual community.

We regard the development of *WaterEngage *as an ongoing collaboration–as features are rolled out, feedback from users informs the next iteration. Successful public engagement programs should engage the public in their own design and development. Focus groups with high school students in North America, for example, have generated feedback that helped shape the development of new tools, such as the ability to post photographs and videos. The email form on *WaterEngage *has prompted users from countries such as Bangladesh and Mexico to share their positive experiences of the *WaterEngage *platform. *WaterEngage *has evolved from initial prototype, to its second iteration, to the third version currently under development [see Additional file 1].

#### Awareness

**Figure 2:**
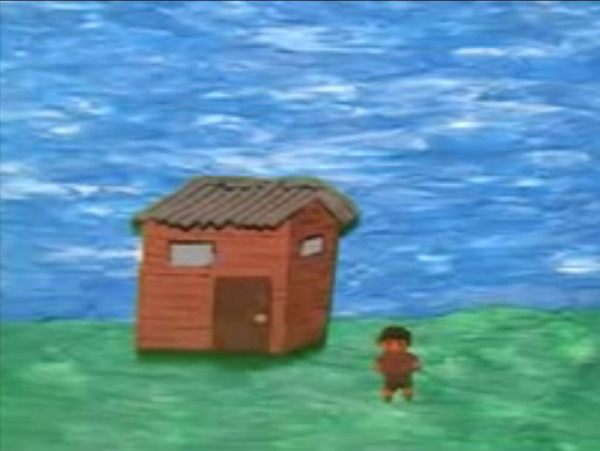
– A student animation describes the socioeconomic impacts of arsenic contamination in Bangladesh.

*WaterEngage *aims to create awareness, fostering informed debate and social action regarding potential solutions to global water problems like scarcity, contamination, and infection. There was no independent process that led to a choice of priority solutions. Instead, a rating and ranking system enables *WaterEngage *users to vote on prominently featured solutions. In this sense,*WaterEngage *offers users a repository of *user-generated *information, videos, and images on numerous water issues (Figure 2).

*WaterEngage *is not a one-way channel for information. Beyond informing, the portal increases awareness of users by providing access to discussion forums and space for individuals and groups to upload or link to their own projects. Users have uploaded their own videos, stories, and animations to share their experiences and knowledge with other youth. *WaterEngage *seeks to challenge youth to start projects that address the water challenges in their communities through the Challenge Fund, awarding small grants to individuals, groups or schools who act to make a difference and positively impact their community's water situation. One project looks at naturally-occurring arsenic, a challenge in both developing countries such as Bangladesh and in industrialized regions such as the province of Nova Scotia, in Canada.

#### Solidarity

These interactive features help to facilitate *WaterEngage*'s second goal, which is to increase solidarity, one of the most important values in global health, encouraging youth to respect and work with distant persons and to develop a global state of mind [19]. With almost one billion people over 15 years of age online, *WaterEngage *has the potential to tap into the power of the Internet to create a community interested in ethical, social, cultural, and scientific issues. This community will promote peer-to-peer information exchange on critical challenges and their potential solutions, from developing local strategies to coordinating youth action, or documenting water problems for a larger public [20]. *WaterEngage *is focused on youth aged 14–24 years to generate creative ideas in the absence of professional pressures.

**Figure 3:**
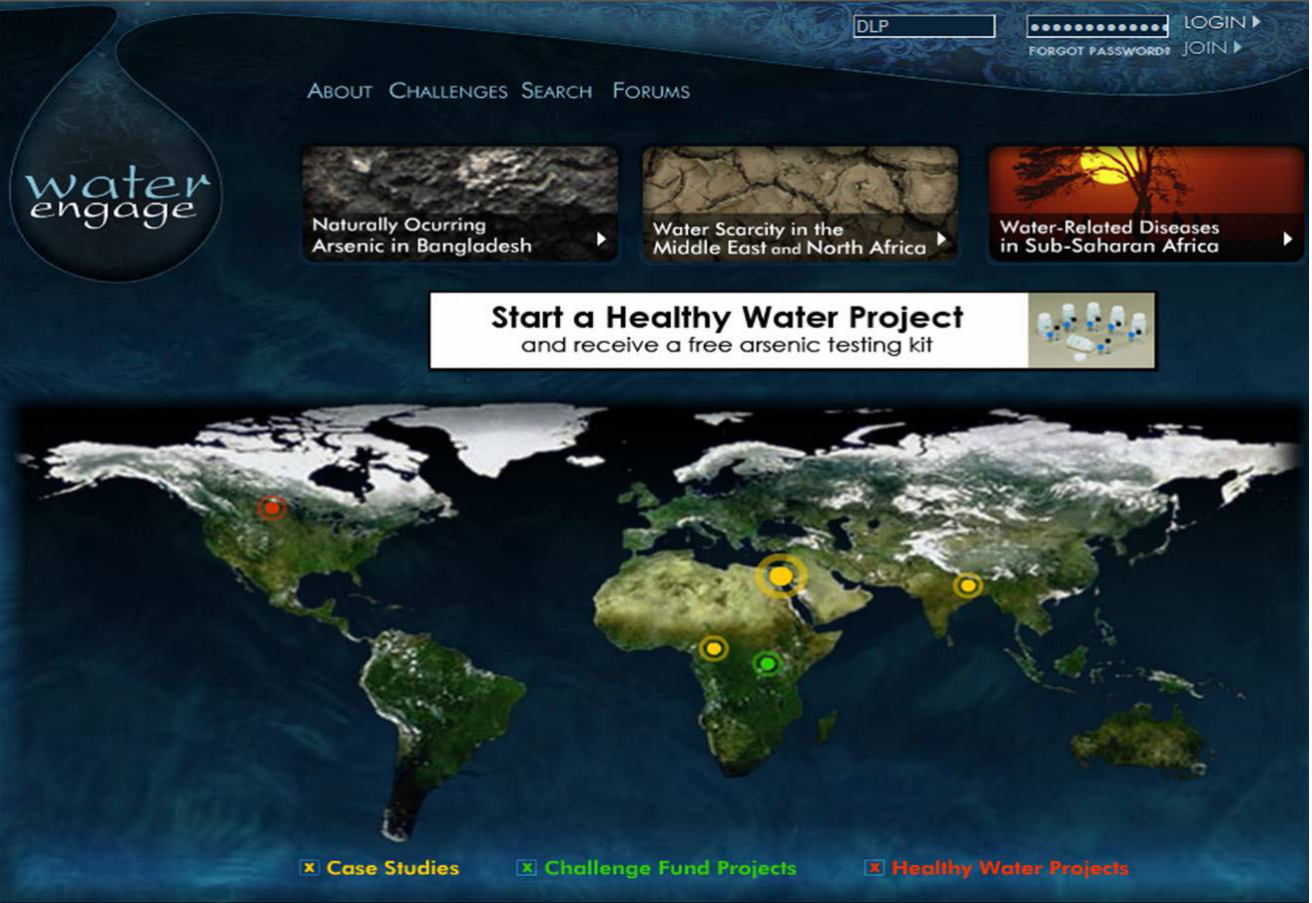
– *WaterEngage *Homepage Version 1.0. Video interviews and online mapping tools.

**Figure 4:**
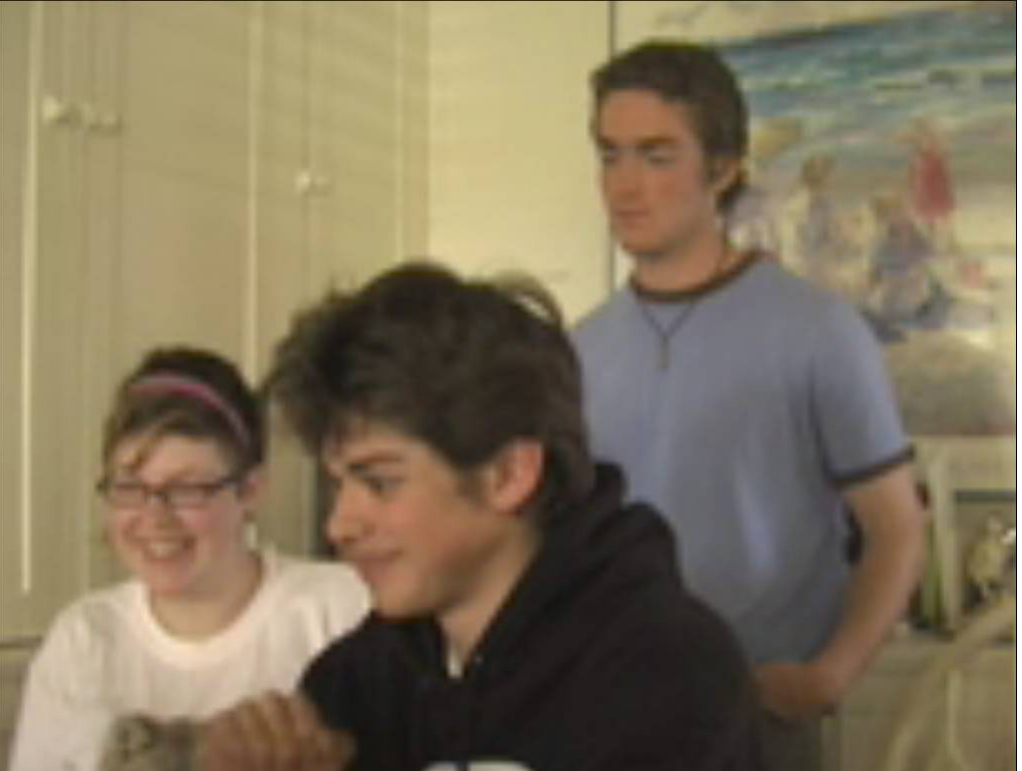
– A video of the interaction between classrooms in San Jose, California, and Dhaka, Bangladesh, in which students have already begun to learn each other's names.

**Figure 5:**
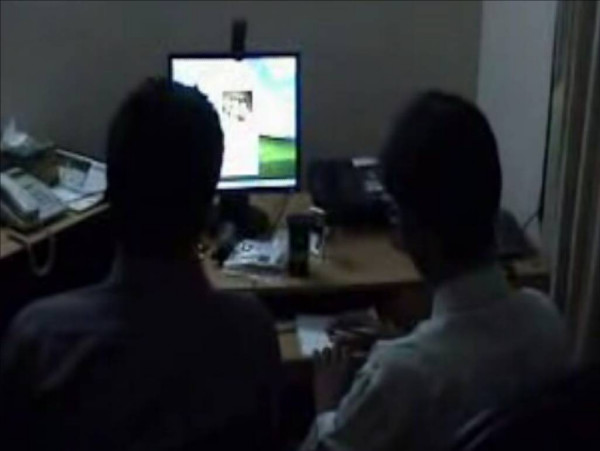
– A video of the interaction between classrooms in San Jose, California, and Dhaka, Bangladesh, in which students have already begun to learn each other's names.

*WaterEngage *also employs social networking capabilities to make it easy for collaborations and friendships to form between young people who share a common interest in water issues. People like to connect with other people, and enabling users to share project results, compare data, and tell their stories builds solidarity with young people in other countries and an awareness of the water challenges they face. To help link youth across borders, the video interviews and online mapping tools (see Figure 3) are used to bring case studies alive. See Figures 4 and 5 to watch a video of the interaction between classrooms in San Jose, California, and Dhaka, Bangladesh, in which students exchange information about the water challenges facing their communities.

#### Technology Development

**Figure 6:**
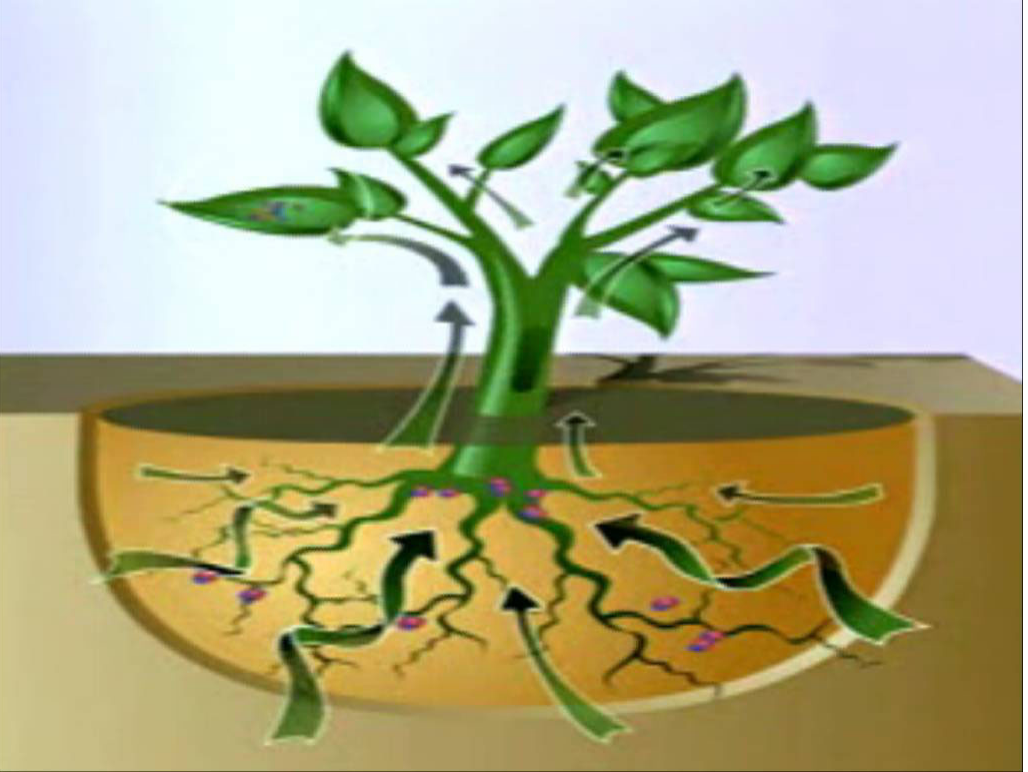
– Videos explaining the newest technologies in water purification and phytoremediation.

**Figure 7:**
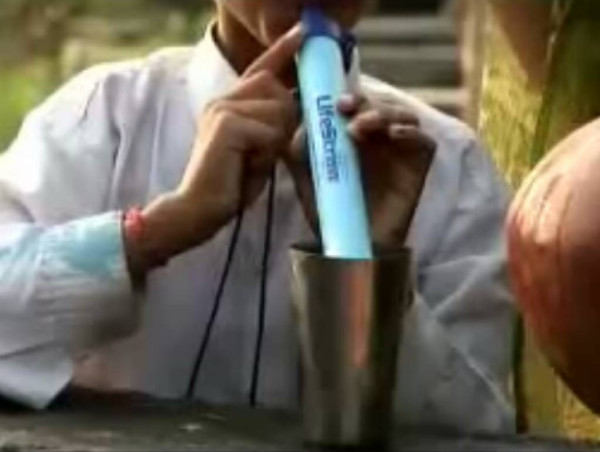
– Videos explaining the newest technologies in water purification and phytoremediation.

Finally, *WaterEngage *aims to engage users in the development of technology-oriented solutions, such as bioremediation, quantum dot-based diagnostics, and nanofilters [21]. *WaterEngage *provides a platform for users to support Southern researchers, scientists and technologists who have solutions that need to be funded and scaled. Creating open venues for knowledge generation and dissemination is a critical part of supporting Southern innovation [22]. See Figures 6 and 7 to watch videos explaining the newest technologies in water purification and phytoremediation.

**Figure 8:**
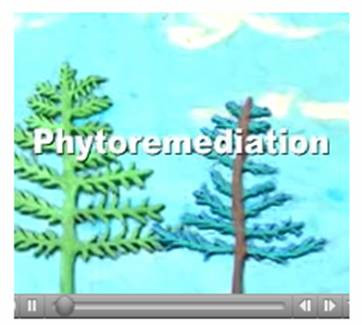
– An animation created by high school students demonstrating phytoremediation.

*WaterEngage *takes the focus from complex scientific issues of emergent technologies, and puts it on water–something everyone can relate to. The *WaterEngage *Challenge Fund encourages and empowers students to innovate and implement their own solutions. While such a technology focus may appear to target the 'scientific citizen,' young *WaterEngage *users have already evidenced the narrowing knowledge gap between the public and scientists by uploading animated videos about scientific concepts [23]. See Figure 8 for an animation created by high school students demonstrating phytoremediation.

### Limitations of WaterEngage

Lack of Internet access in some communities, particularly in developing countries, limits access to online public engagement platforms. In 2004, there were more Internet users in France than all of Africa, and around 30 countries had Internet penetration rates of less than 1% [24]. Poor connectivity infrastructure, low-levels of computer ownership, and the cost of bandwidth present a barrier to online public engagement platforms. Nonetheless, advances in wireless Internet technology and computer hardware is reducing the cost of Internet access and increasing Internet penetration around the world.

A low-bandwidth version of *WaterEngage *is being developed to work with dial-up or intermittent connections. To engage those without access to computers or the Internet, later stages of the project may partner with academia, industry, and civil society organizations to deliver information and collaboration capacity.

*WaterEngag*e's success as a public engagement platform can be measured by analysis of the web usage reports. Metrics include the number of unique users, the level of active participation in the online community, and the percentage of repeat visitors. Although the site has attracted more than 17,000 unique visitors from 85 countries, the community has not yet reached a critical mass of users. Nonetheless, the deep level of participation–defined as posting projects, uploading photographs or commenting in discussion forums–and the frequency of repeat visits indicates the Internet can be an effective tool for public engagement in global health issues. One of the lessons learned is that the platform can partner with other groups mounting campaigns, and thereby provide a useful platform for these groups as well as building awareness of *WaterEngage*. For example, *WaterEngage *is working with the International Water Association to provide a platform for international participants in World Water Monitoring Day to coordinate water monitoring activities.

## Conclusion

This paper outlines basic concepts related to public engagement, giving examples of how this engagement has arisen in the developing world. We also describe a new public engagement platform designed to maximize youth involvement across borders and cultures to solve health challenges. Our next step is to learn from the lessons above from *WaterEngage *and apply them to create *MalariaEngage*. The purpose of *MalariaEngage *is to engage the public in directly funding Southern malaria research and to gain feedback on Northern based-malaria research. By doing so, the public can help achieve fully capitalized and robust malaria research in the developing world so developing world researchers can solve their own problems.

## Competing interests

*WaterEngage *is based on a software platform called Zazengo, which is owned in part by Vicki Saunders and Tom Hadfield.

## Authors' contributions

EC wrote the initial draft of the paper and contributed to all subsequent iterations. HM conducted multiple technical and usability reviews of the *WaterEngage *and MalariaEngage platforms, and co-wrote the final version of the manuscript. KB contributed to all iterations of the paper following the initial draft and co-wrote the final version of the manuscript. VS and TH were involved in developing the *WaterEngage *platform and final editing. DiP co-wrote the initial draft of the paper. DeP and GM contributed to the initial draft of the paper and were involved in generating the concept for the *WaterEngage *platform. AD was involved in developing the *WaterEngage *concept and content, drafting the paper, as well as its revision and final editing. JS was involved in the editing of the final draft. PS was the senior responsible author and involved in all phases of the paper's development.

## Pre-publication history

The pre-publication history for this paper can be accessed here:



## Supplementary Material

Additional file 1 The evolution of *WaterEngage*. Table showing the evolution of WaterEngage. Click here for file
