# Supplementary material for: Public engagement on global health challenges
Source: BMC Public Health. 2008 May 20;8:168. doi: 10.1186/1471-2458-8-168 (PMC2453523; doi:10.1186/1471-2458-8-168)
Supplement: Additional file 1 — The evolution of WaterEngage. Table showing the evolution of WaterEngage. [file 1471-2458-8-168-S1.doc]

**Table 1. The Evolution of WaterEngage**

|  | **Version 1.0**  [www.waterengage.com](http://www.waterengage.com/) | **Version 2.0**  [www.waterengage.com/2.0](http://www.waterengage.com/2.0) | **Version 3.0**  [www.waterengage.org/public](http://www.waterengage.org/public) |
| --- | --- | --- | --- |
| **Model** | Wikipedia | YouTube | MySpace |
| **Content** | “Case studies” are in a wiki for the community to edit. | All content posted as “projects” with an emphasis on video. | Users create profiles, subscribe to and rate other projects, make friend lists. |
| **Technology**  **Advantages** | Uses MediaWiki, a common open source platform (Wikipedia). | Accessible and simple system. Video engages youth, offers a view into countries worldwide. | Opportunity to leverage online social spaces and combine learning with personal connections. |
